# Supplementary material for: Impact of COVID-19 on Breastfeeding among SARS-CoV-2 Infected Pregnant Women: A Single Centre Survey Study
Source: Int J Environ Res Public Health. 2022 Dec 23;20(1):228. doi: 10.3390/ijerph20010228 (PMC9819154; doi:10.3390/ijerph20010228)
Supplement: Supplementary file 1 [file ijerph-20-00228-s001.zip › ijerph-2077991-supplementary.pdf]

Figure S1. Impact of COVID-19 on breastfeeding among SARS-CoV-2 infected pregnant women: a single centre survey study

NAME __________ SURNAME __________ AGE __________

-What is your marital status?

____

-What is your educational level or occupation?

____

-How many pregnancies did you have?

____

-Did you breastfeed your child in previous pregnancies?

____

-How was your baby delivered?

**spontaneous delivery**	**cesarean section**

-Gestational age at delivery:

____

-Lenght of hospedalization:

____

-What was the reason of your hospitalization?

-Do you have any health diseases?

-Did you or your fetus have any complications during the pregnancy?

-Your pregnancy was follow-up mainly by:

**obstetrician**	**midwife**	**hospital**

-Do you feel abandoned, neglected or isolated by your gynecologist because of Sars-COV-2?

-Were you separated from your baby at birth because of Sars-COV-2?

**YES**	**NO**

-If you were separated from your baby, did it affect your ability to breastfeed when you were reunited?

**YES**	**NO**

-Could you have a skin to skin contact in the first hour after the birth?

**YES**	**NO**

-Could you breastfeed your baby?

**YES**	**NO**

-Did you have difficulties in breastfeeding your baby?

**YES**	**NO**

-If you had difficulties, have you been advised to use the breast pump?

**YES**	**NO**

-Did you have difficulties in breastfeeding your baby because of the fear of passing Sars-COV-2 to your son?

**YES**	**NO**

-Did they tell you how to reduce the risk of infecting your baby?

**YES**	**NO**

-Have you been discourage from breastfeed your baby during the Sars-COV-2 infection?

**YES**	**NO**

-Did you have difficulties in breastfeeding your baby because of the symptoms?

**YES**	**NO**

-Did they suggest you to wash your breast?

**YES**	**NO**

-How long did you breastfeed your child?

____

-How and what did you feed your baby in the hospital?

**breastfeeding**	**formula**
**breastfeeding + formula**	**by using the breast pump**

-How and what did you feed your baby at home?

**breastfeeding**	**formula**
**breastfeeding + formula**	**by using the breast pump**

-Did your baby test positive for Sars-COV-2?

**YES**	**NO**

-During the Sars-COV-2 infection you were:

**symptomatic**	**asymptomatic**	**too sick to look after my baby**

-Which symptom have you showed?

-If you decided not to breastfeed your baby, what was the reason?
